# Impact of AI‐based segmentation on PET analyses

**DOI:** 10.1002/alz.093825

**Published:** 2025-01-09

**Authors:** Luc Bracoud, Thomas Cajgfinger, Olivier Rousset, Madhura Ingalhalikar, Chris Conklin, Manja Lehmann, Joel Schaerer, David Scott, Joyce Suhy

**Affiliations:** ^1^ Clario, Inc., Lyon France; ^2^ Clario, Inc., Philadelphia, PA USA; ^3^ Clario, Inc., San Mateo, CA USA; ^4^ Clario, Inc., Munich Germany

## Abstract

**Background:**

Brain MRI segmentation is required for quantitative PET analysis, in order to derive regional uptake and calculate uptake ratio relative to reference regions. FreeSurfer has been a popular method but is being supplanted by faster and more robust AI‐driven methods. The objective of this work is to confirm that the use of Clario’s novel AI segmentation method, whose impact was assessed towards various MRI endpoints, is also valid in the context of PET quantification.

**Method:**

507 subjects from ADNI were selected, including normal controls, subjective memory complainers, early/late MCI and AD subjects. Besides, 210 were selected with available test/re‐test data. For each, a pair of good quality 3DT1 MRI and Amyloid PET scans were available. 3DT1 data were segmented with both FreeSurfer v6 (FS) and Clario’s AI method (AI), and registered to PET space for calculation of Standard Uptake Value (SUV) in a set of cortical regions (frontal, posterior cingulate, lateral parietal and lateral temporal) and reference regions (cerebellar grey and whole cerebellum). SUVR (ratio to reference region) was calculated for both. Results were also converted to Centiloid (CL) units. SUV and SUVR values were compared across segmentation methods via Bland‐Altmann plots and correlated with Pearson’s coefficient. The ability to distinguish between disease severity stages was assessed by ROC analysis using global AUC. Finally, classification as amyloid (Aβ) positive or negative was assessed by setting a cut‐off of 20.1 CL and looking at Cohen’s Kappa.

**Results:**

SUV and SUVR values were highly and significantly correlated between FS and AI methods (r>0.99, p<0.001) and for test/re‐test, for all regions investigated, with a difference that did not exceed 0.41% on average (SD<1.38%). Global AUC was comparable for both methods and for test/retest (average gAUC = 0.62). Aβ classification was near‐perfect across methods (κ = 0.98) and between test/retest data (κFS = 0.97, κAI = 0.99).

**Conclusion:**

Comparing FreeSurfer and Clario’s AI‐based MRI segmentation method, no impact was found on subsequent PET analyses and test/retest performance was equivalent. These results support the use of either method for PET quantification. For future trials, the AI method could either be used as the primary method, or as a back‐up when traditional FS segmentation fails.

